# High-performing cross-dataset machine learning reveals robust microbiota alteration in secondary apical periodontitis

**DOI:** 10.3389/fcimb.2024.1393108

**Published:** 2024-06-21

**Authors:** Hao Li, Jiehang Li, Jiani Hu, Jionglin Chen, Wei Zhou

**Affiliations:** ^1^ Department of Endodontics, Shanghai Ninth People’s Hospital, Shanghai Jiao Tong University School of Medicine, College of Stomatology, Shanghai Jiao Tong University, Shanghai, China; ^2^ National Center for Stomatology, National Clinical Research Center for Oral Diseases, Shanghai Key Laboratory of Stomatology, Shanghai, China; ^3^ Research and Development Department, Beijing Xunzhu Biotechnology Co. Ltd., Beijing, China; ^4^ School of Chemistry Molecular Biosciences, The University of Queensland, Brisbane, QLD, Australia

**Keywords:** oral microbiota, apical periodontitis, meta-analysis, machine learning, secondary apical periodontitis, oral microbiome

## Abstract

Multiple research groups have consistently underscored the intricate interplay between the microbiome and apical periodontitis. However, the presence of variability in experimental design and quantitative assessment have added a layer of complexity, making it challenging to comprehensively assess the relationship. Through an unbiased methodological refinement analysis, we re-analyzed 4 microbiota studies including 120 apical samples from infected teeth (with/without root canal treatment), healthy teeth, using meta-analysis and machine learning. With high-performing machine-learning models, we discover disease signatures of related species and enriched metabolic pathways, expanded understanding of apical periodontitis with potential therapeutic implications. Our approach employs uniform computational tools across datasets to leverage statistical power and define a reproducible signal potentially linked to the development of secondary apical periodontitis (SAP).

## 1 Introduction

Apical periodontitis (AP), a distinctive endodontic disease characterized by inflammatory lesions around the tooth apical, is primarily attributed to microbial intrusion into the root canal system (Kakehashi et al., 1965; Nair, 1997). Typically stemming from untreated dental caries, this infection leads to symptomatic manifestations and, in severe cases, life-threatening abscesses. The standard approach to AP treatment involves antibiotic therapy and either root canal treatment (RCT), also known as endodontic therapy, or extraction of the affected tooth to eradicate the source of infection (Siqueira et al., 2000).

Though many cases resolve with appropriate root canal treatment, known as primary apical periodontitis (PAP), clinical studies reported an alarming number of cases with persistent case of AP, characterized by persistent inflammation, known as secondary apical periodontitis (SAP) or post-treatment apical periodontitis (Sundqvist et al., 1998; Siqueira et al., 2014). A substantial proportion (30–65%) of root-filled teeth may exhibit radiographic evidence of secondary apical periodontitis even when treatment has followed proper standards (Sjögren et al., 1990; Ekelund et al., 2003; Siqueira and Rôças, 2013; Segura-Egea et al., 2015). SAP disease is closely linked with intraradicular infection, caused by bacteria that resist treatment and lead to ongoing periradicular inflammation. These bacteria are often found in hard-to-reach areas like the root canal’s apical part, lateral canals, apical ramifications, isthmuses, and dentinal tubules, where they access nutrients from surrounding tissues (Ricucci et al., 2009; Mombelli and Décaillet, 2011; Rôças and Siqueira, 2012; Vieira et al., 2012). Endodontic treatment of teeth affected by apical periodontitis typically exhibits a reduced success rate. This may necessitate further interventions, such as endodontic microsurgery or apical microsurgery, and in certain cases, tooth extraction might be required to resolve the issue (Ricucci et al., 2011; Siqueira et al., 2014). The persistent infection caused by SAP can lead to an increased risk of various systemic diseases, particularly cardiovascular diseases and diabetes (Segura-Egea et al., 2015).

Previous investigations utilizing broad-range culture and 16S rRNA sequencing have identified a variety of relevant species into the microbial communities associated with necrotic root canals, primarily strict anaerobic bacteria from Peptostreptococcus, Prevotella, Porphyromonas, Fusobacterium, Eubacterium, and Actinomyces, along with facultative anaerobic Streptococci (Sakamoto et al., 2006; Tatikonda et al., 2017). Notably, the presence of these pathogens has been linked to primary apical periodontitis; whereas, secondary apical periodontitis exhibits distinct microbial populations, predominantly Gram-positive facultative anaerobes like *Streptococcus*, *Lactobacillus*, and *Enterococcus* (Siqueira and Rôças, 2005; Siqueira et al., 2016; Qian et al., 2019). The emergence of *Enterococcus faecalis* is identified as a frequently isolated bacterium in root-filled teeth, and has drawn attention due to its biofilm establish resistance against many conventional antimicrobial agents and root canal sealer (Peciuliene et al., 2001; Johnson et al., 2006; Wang et al., 2021). However, not all cases of SAP exhibit the presence of *E. faecalis*, indicating the existence of other potential contributing microorganisms.

Despite previous efforts to investigate the impact of the microbiome on AP, further analyses were hindered to identify reproducible signals across studies (Kumar et al., 2012; Vengerfeldt et al., 2014; Siqueira et al., 2016; Bouillaguet et al., 2018; Qian et al., 2019), due to inconsistencies in experimental settings and a lack of common quantitative definitions, commonly referred to as “reproducibility crisis” (Baker, 2016). Interpretation of the canal bacterial community’s effects was complicated by technical and biological inconsistencies. Even though exploring similar variables (bacterial communities collected) and outcomes (disease stages: health, SAP, or PAP), these studies varied in inconsistent control settings and rooted in population differences. For instance, Zhang et al. (2022) studied diseased tooth canal using supragingival samples from healthy tooth as control groups, whereas Bouillaguet et al. (2018) applied dentin from diseased tooth as controls. Control samples from other studies such as Vengerfeldt et al. (2014); Qian et al. (2019) were variable from canal surface to root, exacerbating complexities. Experiment design involved variability in sample tissues, population biases, or control group configurations, making the core bacterial communities associated with primary and secondary Apical Periodontitis (AP) remain refuted. As a consequence, scientific reanalysis is essential to systematically address these inconsistencies in an unbiased manner, which benefits a robust foundation for AP progression (Gurevitch et al., 2018).

In the current field of microbiome research, deep learning methods across datasets are considered effective means to acquire profound microbiome knowledge. Meta-analysis systematically quantified and mitigated technical variation and contamination. These methods can handle large-scale microbiome data and discover patterns and regularities hidden within the data. In this study, we place particular emphasis on the significance of deep learning for extracting knowledge from cross-dataset apical AP microbiomes, revealing the pivotal microbial taxa and functional pathways linked to PAP and SAP, thereby.

Here we present the meta-analysis and machine learning of 16S rRNA sequencing-based studies investigating the effect of apical microbiome on apical periodontitis progress. Rigorous measures were taken to eliminate observed batch effects from data sources and exclude data from inappropriate control groups. What is more, machine learning further identify microbial signatures both phylogenetic and pathway levels, distinguishing among different disease stages. High-predictive machine-learning models (AUROC *>* 0.95, AUPR *>* 0.9) unveiled signatures that predict various disease types, demonstrated that phylogenetic and gene-centric transformations contribute to shaping the overall disease landscape. Finer systematic analysis unveiled that, beyond the previously acknowledged influence of *Enterococcus faecalis*, *Cutibacterium acnes* and *Delftia acidovorans* may also be implicated in the occurrence of secondary apical periodontitis. The phosphotransferase system and peptidoglycan biosynthesis pathway were enriched among different apical periodontitis stages. This revelation extends our comprehension of apical periodontitis, holds the potential to serve as a foundation for targeted therapeutic interventions.

## 2 Materials and methods

### 2.1 Study selection

The following all encompassing search term was entered into PubMed and the NCBI Sequence Read Archive (SRA) in Oct 2023 to generate an unbiased representation of studies studying the relationship between bacteria community and apical periodontitis.

“‘apical periodontitis”[All Fields] AND “microbiome”[All Fields] OR “apical periodontitis”[All Fields] AND “microbiota”[All Fields] OR “apical periodontitis”[All Fields] AND “bacteria community”[All Fields] “

Among these, we identified 7 studies as sequencing-based research. We further examined the sampling and sequencing methods, selected only paired-end sequencing data sets, and removed samples from the maxillofacial region or samples that had not been externally sterilized during the sampling. Of these 7 studies, 4 datasets(SRA ID: SRP075560, SRP121389, ERP108053, SRP361111) from Siqueira et al. (2016); Bouillaguet et al. (2018); Qian et al. (2019); Zhang et al. (2022) encompass a collection of 224 samples that include 110 dentin and periapical microbiota from teeth with primary apical periodontitis, 95 dentin and periapical microbiota samples with secondary apical periodontitis, and 19 periapical microbiota samples from healthy molar. we filtered out 104 samples from the dentin, thereby ensuring that the characteristics we ultimately observed were associated exclusively with the disease process, rather than with the tissue type. All SAP samples were identified by imaging evidence, and none of the selected samples had been on antibiotics for two weeks.

### 2.2 Data retrieval and ASV picking

120 final samples were derived via V3-V4 16S rRNA amplicon sequencing on Illumina-platform. Raw reads were downloaded for 120 samples from the NCBI Sequence Read Archive (SRA), then were filtered on quality in qiime2 2023.9 (Bolyen et al., 2019). Pair reads were aligned and denoised using deblur following parameters –p-trim-length 200/–p-min-reads 10. A total of 6,636 ASVs were observed across 110 samples, those ASV numbers varies from data sources (**Supplementary Figure S1**). The feature table was annotated using naive-bayes classifer trained on eHOMD v15.23 from (Escapa et al., 2018) release in qiime2. The ASV feature table was converted to a biom file and processed with PIRCRUSt (Langille et al., 2013) for ko and ec recognition. Tree based on feature table was generated using qiime2 fasttree pipeline. We further refined the dataset by filtering out near-zero variance ASVs using the ‘nearZeroVar’ function from the ‘caret’ package and ASVs not presenting in at least 3 samples with at least a total of 10 reads with ‘Confidence.Filter’ function from ‘MicrobeR’ package, resulting in 2063 ASVs. Additionally, we filtered out datasets whose depth is less than 1000 reads. This process resulted in a final feature table containing 1740 ASVs. In order to demonstrate our data processing, we also set up 2 group with data filter1 presenting in at least 1 sample with at least a total of 10 reads, and data filter2 presenting in at least 3 samples with at least a total of 500 reads.

### 2.3 PVCA and batch effect elimination

Raw counts were normalized using limma (Ritchie et al., 2015) package and then use sva package (Leek et al., 2012) to reduce batch effect with command (ComBat(dat=voomdata, batch=BatchVariable, mod=NULL, par.prior=TRUE), in which ‘BatchVariable’ refers to data sources. Principal variance components analysis was used to quantified these changes between raw count data and SNM-corrected data using pvca (Bushel, 2024) package in R.

### 2.4 Diversity analysis

For analysis on a per study basis, samples were rarefied to 5000 depth samples for generating alpha diversity metrics. The diversity and estimateR functions of Vegan (Dixon, 2003) were used to generate Shannon’s diversity index (log base e) and Chao1 estimates respectively and Picante (Kembel et al., 2010) was used to generate Faith’s phylogenetic distance. UniFrac and Jensen-Shannon divergence were calculated using the parallel-enabled distance function of Phyloseq (McMURDIE and Holmes, 2012) on subsampled proportional abundances. Bray-Curtis dissimilarity was also calculated (vegdist, Vegan) on subsampled proportional abundances. The CLR Euclidean distance was calculated by carrying out a centered log2-ratio transformation (Make.CLR, MicrobeR) with count zero multiplicative replacement [zCompositions (Palarea-Albaladejo and Martín-Fernández, 2015)] followed by calculating the Euclidean distance (dist, base). The PhILR Euclidian distance was calculated by first carrying out the phylogenetic isometric log ratio transformation [philr, PhILR (Silverman et al., 2017)] and calculating the distance matrix as before. Principal coordinates analysis was carried out using the pcoa function of APE (Paradis and Schliep, 2019). ADONIS calculations were carried out using adonis2 in Vegan on each distance/dissimilarity metric.

### 2.5 Models selection and evaluation

Our study evaluated machine learning models commonly used in genetics, including Random Forest (RF), Support Vector Machine (SVM) with radial and linear kernels, and Logit Boost (LB). RF is known for its robust performance in datasets with a high feature-to-sample ratio and is effective in handling weak predictors and complex interactions. SVM applies kernel functions for robust modeling, even with outliers. LB, part of the boosting family of algorithms, creates strong classifiers by combining multiple weak ones, known for improved accuracy and robustness. Analyses were performed in R Version 4.3.1 using the ‘caret’ package.

Models were built 5 times cross validation by random data splitting, training of the models, making predictions and recording of accuracies after each run using the caret packages for the R statistical environment (Kuhn, 2008). Cross-validation and parallel processing were enabled by the inclusion of a train control parameter. Analysis of variance, at significance alpha value of 0.05 was used to analyze the differences in mean accuracy between the models.

To enhance model performance and interpretability, our approach incorporated a rigorous search for key features within the dataset. This process involved analyzing a wide array of potential predictors, identifying those with the most significant impact on the predictive accuracy of our models. The identification of these key features is critical, especially in complex datasets, as it aids in refining the model and focusing on the most relevant variables. Since our classification target here has three categories, we need to introduce the multiROC package (Pérez-Fernández et al., 2021). The performance of our models was evaluated using two metrics: Area Under the Receiver Operating Characteristic (AUROC), which measures the model’s ability to distinguish between classes, and Area Under the Precision-Recall Curve (AUPR), important for assessing models in imbalanced datasets by evaluating precision and recall. Macro-average ROC/AUC was chosen in this study to describe each model, and it was calculated by averaging all groups results (one vs rest) and linear interpolation was used between points of ROC.

The number of predictor variables was determined by selecting the point of saturation in minimizing error rate and selecting the features based on ranked MeanDecreaseGINI, all other features were excluded from the model. Mtry and Ntree were left as default values (Mtry = sqrt(Nfeatures) and Ntree = 500).

### 2.6 Subsampling for class imbalances

Our exploration of clinical outcome classes revealed significant class imbalances (as shown in **Supplementary Figure S1**, **Supplementary Table S4**). Such imbalances can lead to models with poor class-specific performance, as the training process tends to favor patterns associated with larger classes (Velez et al., 2007). To mitigate the effects of this imbalance on our trained model, we employed *post hoc* sampling approaches (Kuhn et al., 2013). Given the considerably low number of samples in the smallest class, we opted for up-sampling, utilizing methods available in the R environment. The Logit Boost learning method, along with 5-fold cross-validation, was employed to evaluate the effectiveness of our resampling approach.

## 3 Results

### 3.1 Study selection and characteristics

63 unique studies were retrieved by our search methodology. Among these, 7 studies were identified as sequencing-based and met the eligibility criteria for inclusion in our meta-analysis. Of these 7 studies, 3 lacked public, accessible sequencing data or metadata sufficient for pairing sequencing. As an illustrative example highlighting the difficult acquiring data post-publication, we attempted to contact the corresponding author via email for sequencing data without any response. This left 4 studies for inclusion in our meta-analysis, encompassing a collection of 120 samples (Siqueira et al., 2016; Bouillaguet et al., 2018; Qian et al., 2019; Zhang et al., 2022).

**Figure 1:**
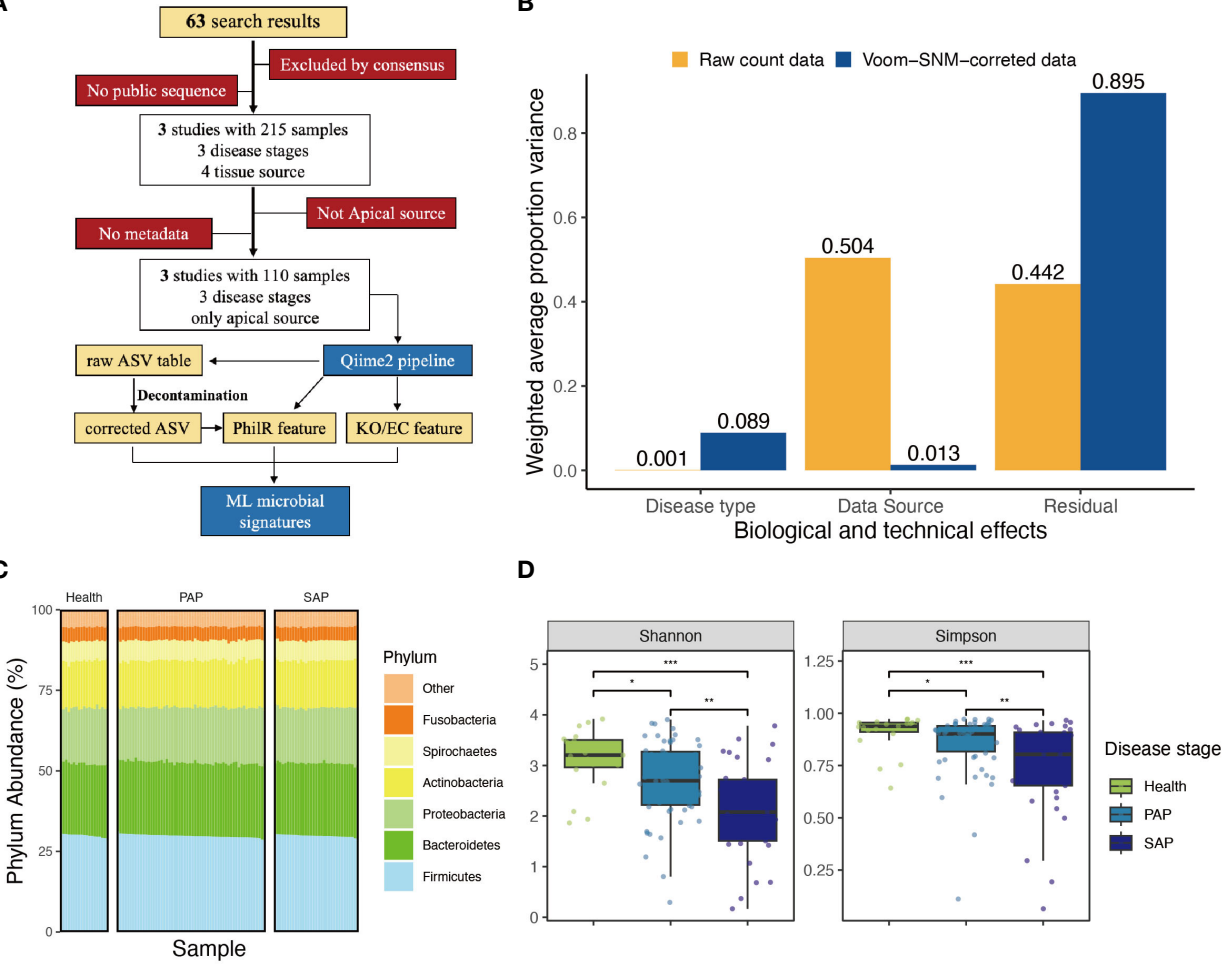
Approach and overall findings of microbiome analysis in AP. **(A)** CONSORT-style diagram showing quality control processing and the number of remaining samples. **(B)** Principal variance components analysis of raw taxonomical count data and Voom-SNM corrected data, showing the variance sources before and after data correction. **(C)** Phylum abundance of each sample, group by disease stage, color refers to different phylums. **(D)** ;Measures of alpha diversity including Shannon’s diversity, and Shimpson diversity demonstrate inconsistent effects of disease type, complete figure is shown in **Supplementary Figure S12**, the signal indicating the statistic difference (***p ≤ 0.001; **0.001 *<* p ≤ 0.01; *0.01 *<* p ≤ 0.05, NS, 0.5 *<* p).

The afflicted samples were limited to those presenting radiographic evidence of periapical lesions, and excluding any with crown damage, severe oral health conditions, or antibiotic treatment within one month prior to extraction. Contamination from saliva was minimized by strictly experimental condition in each study. To establish robust controls and focus our comparison on the apical, we filtered out 105 samples from the dentin, thereby ensuring that the characteristics we ultimately observed were associated exclusively with the disease process, rather than with the tooth structure itself (**Figure 1A**). From the initial 6663 ASVs identified from the sequenced data, we got 1740 ASVs which are present in at least 3 samples with at least a total of 10 reads. We examined the distribution of these remaining ASVs in different study sources (**Supplementary Figure S1**) and further analyzed their community composition below. We also checked the current data structure and found that there are two datasets with small sample sizes (¡20), and the number between different disease stages is unbalanced. Therefore, we integrate them together for processing instead of applying independent validation on each dataset latter.

### 3.2 Selected studies showed biased batch effort

Sequencing data obtained from different instruments and studies often exhibit significant batch effects and contamination, especially in microbiota (Salter et al., 2014; Glassing et al., 2016; Poore et al., 2020). We applied PCoA to visualize the hidden batch effect in each study by comparing their community compositions. We first calculated common metrics for beta diversity for PCoA through Bray-Curtis dissimilarity, weighted/unweighted UniFrac, Jensen-Shannon divergence, PhILR Euclidean distance, and CLR Euclidean distance. Due to matrix sparsity, significant distance saturation was observed when all studies were aggregated (**Supplementary Figure S6**), so only 3 metrics were employed: CLR-Euclidean, unweighted UniFrac, PhILR Euclidean, and associated scree plot. We then employed visualization strategies and statistically tested the effect of data source on community composition. Clear visual clustering independent of study was observed by using principal coordinates analysis of all distance metrics, and ADONIS analysis showed significant difference between data sources (**Supplementary Table S1**).

Given the clear evidence the existed batch effort in each study, we implemented a pipeline to eliminate observed batch effort in all datasets. Subsequently, we applied supervised normalization (SNM) to reduce noise from data sources variability, while preserving and highlighting the biological variability of interest. Principal Variance Components Analysis (PVCA) demonstrated that SNM-correction mitigated batch effects in main technical variance from 0.504 to 0.013, and enhance the biological signal, specifically the ‘disease type’ from 0.001 to 0.089. Meanwhile, 89.5% of the residual variance in SNM-corrected data suggests the limitations of differential analysis in addressing this dataset. To extract relevant features, we need to employ more sophisticated models, indicating the necessity of applying machine learning approaches (**Figure 1B**; **Supplementary Figure S3**).

### 3.3 Apical periodontitis reduce microbial diversity

**Figure 2:**
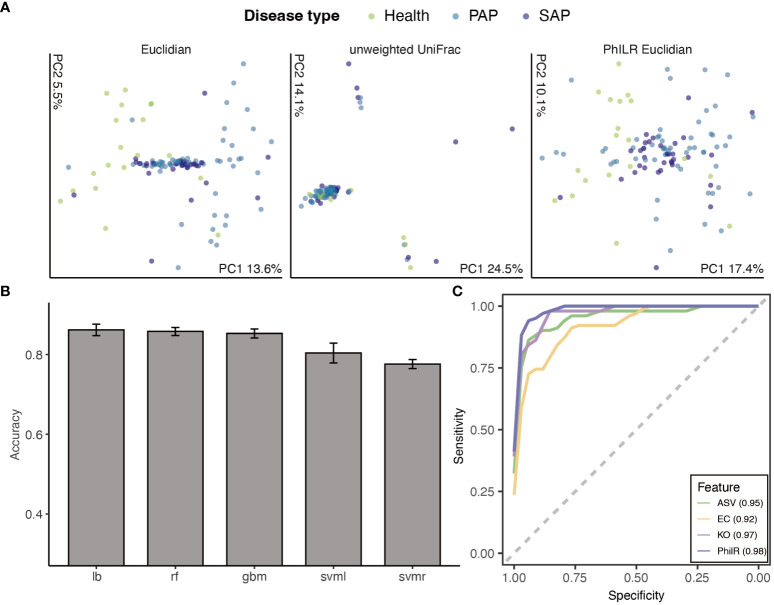
Machine learning approaches to classify disease stages. **(A)** Principal Coordinate Analysis of Samples by disease stage. Ordination, where compositionally related samples are co-localized, provides clear visual evidence for a significant effect of disease stages(p ¡ 0.001 ADONIS). **(B)** Accuracy of different models applied to classification tasks, ranked by mean accuracy. Models included random forest (RF), support vector machine (SVM) (radial and linear kernels), gradient boosting machine(GBM) and logit boost (LB). **(C)** Receiver operator curves for ASVs, phylogenetic node balances (PhILR), KEGG orthologies (KO) and Enzyme commission(EC) trained Logit boost models. The integrated area under the receiver operator curve (AUROC) for each model is provided in the bottom right corner, with higher AUROC values indicating better model performance. Certain AUROC of each class are provided in **Supplementary Table**. Lines are colored by representative method.

SNM-corrected data was applied to generate an unbiased distribution of periapical microbial community (**Figure 2A**), which is dominated by Firmicutes, Bacteroidetes, Proteobacteria, and Actinobacteria (**Figure 1C**). Within the Firmicutes, orders such as Lactobacillales have been implicated in the fermentation of dietary carbohydrates, a process integral to oral microbial homeostasis. Similarly, the abundance of Bacteroidetes, particularly the Bacteroidia class, aligns with recent insights into their enzymatic capabilities and their potential to degrade polysaccharides, a function essential in the oral ecological balance (**Supplementary Figure S4**).

Then, we estimate whether the biodiversity of oral microbial environment relates to AP development. We calculated common metrics for alpha diversity, Chao1 richness, Shannon’s diversity, and Faith’s phylogenetic diversity. Taking all studies into consideration, it was observed that the alpha diversity of the periapical microbiota consistently decreased with the progression of apical periodontitis (**Figure 1D**; **Supplementary Figure S11**) Significant differences were found in the ACE and Chao1 richness indices between healthy samples and primary apical periodontitis samples (p-values < 0.001). This suggests that the occurrence of apical periodontitis is associated with a reduction in the alpha diversity of the periapical microbial community. Furthermore, significant differences (p-values < 0.01) in Shannon and Simpson richness indices were observed between PAP and SAP samples. This is likely attributed to the effects of root canal treatment and indicates that in apical periodontitis, a small number of pathogenic bacteria play a dominant role in disease progression.

We next re-tested the relationship between apical periodontitis and beta diversity, i.e. community composition. Multiple distance metrics acquired from statistical testing via ADONIS (analysis of variance using distance matrices) are used for principal coordinates analysis. All distance demonstrated a significant correlation between disease stage and community composition (p ¡0.01, ADONIS, **Supplementary Table S1**), albeit with the variance explained ranging from 0.032 to 0.121 (R square, **Supplementary Table S1**). In summary, both alpha and beta biodiversity is highly correlated to AP development, yet principle coordinate analysis cannot provide simple prediction accordingly.

### 3.4 Predictive microbial responses to AP stage

Next, we attempted to apply machine learning on the corrected ASV table, in order to create a predictive model of AP stages. To provide better interpretability and reduce the complexity of model training, we removed ASVs with near-zero changes among all samples and discarded any ASVs un-annotated at the genus level, which left 1740 ASVs. We selected five machine learning (ML) methods—random forest (RF), support vector machine (SVM) with radial and linear kernels, gradient boosting machine (GBM), and logit boost (LB)—to cover a spectrum of approaches commonly employed in bioinformatics for analyzing complex datasets. These methods are renowned for their capacity to handle high-dimensional data and discern intricate variable interrelationships. By integrating these methods, our aim was to construct a robust, accurate, and interpretable predictive model for AP stages, a standard practice in microbiomics for classification tasks. Datasets were randomly divided into 7:3 for training and test sets, with training details in Methods. Initially, class imbalances in dataset (19:58:43 in health:PAP: SAP) resulted in poor performance with average accuracy of 5 cross-validations under 0.5 in all models. Thus, we upsampled cases from the minority classes with replacement until each class had approximately the same number, and retrained models.

Meanwhile, we employ other 3 feature representation methods on raw 6663 ASVs to enrich the interpretability of the data and elucidate the functioning and potential impacts of microbial communities. The PhilR (Phylogenetic Isometric Log-Ratio) approach transforms species abundance data into balanced evolutionary ratios using phylogenetic trees, providing a more stable and interpretable feature space that captures the true biological signals within the evolutionary context. KEGG Orthology (KO) features, derived from the KEGG database (Kanehisa et al., 2017), annotate the gene content of the microbiome to identify genes associated with known metabolic pathways and biological processes, revealing the functional potential of microbial communities. Enzyme Commission (EC) numbers annotate genes encoding enzymes in the microbiome genomes, reflecting the enzymatic repertoire and metabolic activities present within the community.

After applying up-sampling to all four features (ASV/PhilR/KO/EC), we compared the aforementioned performance metrics. Upon 3 repetitions, methods such as Gradient Boosting Machine (gbm), Random Forest (rf), and Logistic Regression (lb) demonstrated higher average accuracy. Furthermore, we depicted multi-class ROC and PR curves and computed the corresponding AUROC and AUPR values. Notably, gbm and lb showed higher values for these metrics (**Figure 2B**). Likely due to data sparsity and interspecies variation in ASV content, the model trained on ASVs counts had a high AUROC(AUROC = 0.897 in gbm model), indicating a good separation between classes, but shown lower Area Under the Precision-Recall Curve AUPR(AUPR *<* ¡0.85, **Supplementary Figure S10**), suggesting that the model’s performance on predicting the minority class was not as strong, particularly in cases where the positive class is less prevalent.

Logistic Regression showed to be the best-performing model according to 5-fold cross-validation accuracy, followed by gbm and svm with a radial basis function kernel, respectively (**Figure 2B**). We then employed a Logistic Regression classifier to define reliable biomarkers of AP stage based on a training set consist 70% of whole dataset. This model exhibits the highest performance on the philR features (multiclass AUROC = 0.967, **Figure 2C**), indicating that the additional evolutionary node information confers enhanced its predictive power .(**Figure 2C**) 10-fold cross validation was applied to determine the optimal number of each features included in the model required to minimize error rates. We noted that even with as few as 14 ASVs, 0.3% classification error rates could be obtained, emphasizing the high predictive power of the top features. (**Supplementary Figure S5**) To visualize these, a phylogenetic tree of the 14 most informative ASVs was created. Among these ASVs, we scrutinized the abundance of the Lactobacillus genus, a group frequently encountered in food and cause contamination in microbiota analysis, ensuring that the contamination from food-borne bacteria was not shown in our features. To validate the result under varying data cleaning processes, we also compare the number of key ASVs from RF model training on dataset with different filtering criteria (**Supplementary Figure S12**). 10 key ASVs were commonly recognized from 3 filtered data indicating the robustness of our data processing.

**Figure 3:**
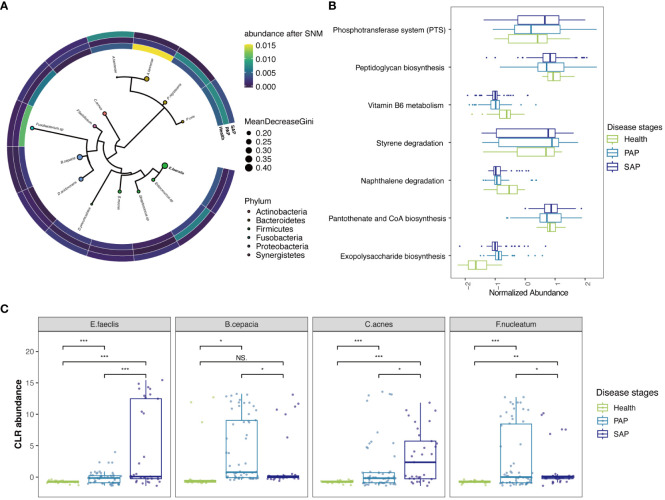
Reproducible Signature of the AP-Associated Apical Microbiome. **(A)** Phylogenetic tree of informative ASVs (n = 14) demonstrates highly informative clades. Size of circle correlates with mean decrease in GINI coefficient and circles are colored by phylum. The heatmap around phylogenetic tree showed CLR abundance of the ASVs in each stage, continuous colors from yellow to blue refer to CLR abundance from high to low. **(B)** key KO features enriched and their abundance, the y-axis corresponds to KO features. Boxlots are colored by disease stages, those features are ranked by p-values. Raw data is provided in **Supplementary Table S3**. **(C)** CLR abundances for selected key species *E. faeclis, B. cepacia, C. acnes* and *F. nucleatum* in 3 disease stages (***p ≤ 0.001; **0.001 *<* p ≤ 0.01; *0.01 *<* p ≤ 0.05, NS, 0.5 *<* p).

The most predictive ASV, as indicated by the mean decrease in GINI coefficient (**Figure 3A**), belonged to *Enterococcus faecalis*, which also showed significant differences in raw ASV counts (**Figure 3C**). It is a commonly reported multidrug-resistant pathogen that is prevalent in secondary apical periodontitis (SAP) in previous studies (Peciuliene et al., 2001; Johnson et al., 2006; Wang et al., 2012). However, its role has been controversial in differential 16SrRNA-sequencing-based analysis (Bouillaguet et al., 2018; Qian et al., 2019), and our analysis has now provided further confirmation across datasets. It is recognized that *E.faecalis* serves as the strongest indicator signal for distinguishing different stages of apical periodontitis, especially in SAP, where it exhibits significantly high abundance (**Figures 3B, C**). *Cutibacterium acnes* (*C.acnes*) and *Delftia acidovorans*(*D.acidovorans*) also displayed a similar characteristic of a substantial increase in abundance in SAP. (**Figure 3C**) This suggests that treatment failures in root canal therapy may be closely related to the persistent presence of those bacteria, which could be responsible for the development of SAP during the root canal filling process. In recent transcriptome-based differential analyses of primary and secondary AP, *C.acnes* unexpectedly contributed the largest number of differential genes at the transcriptomic level (Pinheiro et al., 2022). This specie was also highlighted as an active member of the persistent community in a RT-PCR based study (Nardello et al., 2020), and popular in root canal after chemomechanical procedures (Nardello et al., 2022). Remarkably, our batch-corrected data endorses *D. acidovorans* as a crucial contributor to the progression of SAP, as proposed in Anderson et al. (2012), while some studies have recognized its protective role in root caries (Abram et al., 2022).

Prevotellaceae family, with the highest proportion in ASVs features, was once again highlighted in PhILR features. (**Figure 3A**; **Supplementary Figure S5**) Previous studies have also emphasized the role of Prevotellaceae in periodontal inflammation (Könönen et al., 2022). Within this family, Prevotella nigrescens and Prevotella oris, initially isolated from periodontal pockets and considered important pathogenic bacteria in periodontal inflammation, were found to have a significant impact on the progression of apical periodontitis in our analysis. Notably, *Alloprevotella tannerae* from the Prevotellaceae family, previously reported to induce dentin caries, showed an increase in prevalence in primary apical periodontitis but a reduction in SAP. This suggests that these bacteria play a role in the pathogenesis of primary apical periodontitis, but they are effectively cleared by root canal treatment. Similarly, bacteria such as *Burkholderia cepacia* and *Fusobacterium nucleatum*, which can be effectively managed by root canal treatment, were enriched in PAP and have been previously detected in periapical abscess samples (Pinheiro et al., 2022). The role of *Fusobacterium* has been emphasized in primary infections at the transcriptomic level in a recent study, in which the *Fusobacterium nucleatum* carries over 10 antibiotic resistance genes homologs in multiple cases (Pinheiro et al., 2022).

Utilizing KO and EC features, we were able to perform an enrichment pathway analysis that honed in on particular metabolic pathways of interest. Our findings notably spotlight the phosphotransferase system (PTS), a pathway familiarly documented in biofilm of Streptococcus and Enterococcus species, which is intricately connected to the etiology of dental caries (Suriyanarayanan et al., 2018) (**Figure 3B**; **Supplementary Table S2**). This discovery is in harmony with our cross-validation results concerning Streptococcus and Enterococcus in the ASVs feature. Complementing this, our data derived from EC features also showed a significant enrichment in fructose and mannose metabolism (**Supplementary Table S3**). These pathway-based analyses collectively underscore the significance of carbohydrate metabolism pathways, highlighting their pivotal role in the pathogenesis of AP and indicating potential biological mechanisms.

## 4 Discussion

The intricate interplay between the microbiome and AP has long been a subject of scientific intrigue. However, the presence of variability in experimental design and quantitative assessment has added layers of complexity, obscuring a comprehensive understanding of this dental malady. In this exploratory voyage, we embarked on a methodological refinement analysis, reexamining four microbiota studies encompassing 120 tooth samples from afflicted teeth (with or without root canal treatment) and healthy teeth. Our relentless quest aimed to eliminate batch effects, banishing data from incongruous control groups. Employing high-predictive machine learning models, we unveiled microbial signatures capable of predicting diverse disease types. In addition, our odyssey unearthed hitherto less-recognized culprits in the genesis of secondary apical periodontitis, offering tantalizing prospects for targeted therapeutic interventions. Furthermore, our analysis identified pathways, such as the phosphotransferase system and peptidoglycan biosynthesis, enriched during the progression of AP. This revelation extends the boundaries of our comprehension of AP and lays the foundation for precision-focused therapeutic strategies.

Our comprehensive analysis has harmonized various datasets, aligning control setups and analysis standards. As previously reported, datasets from different studies included various bias from experimental contamination or batch effect (Salter et al., 2014; Glassing et al., 2016; Poore et al., 2020). We have uncovered significant technical and experimental differences existing in previous study on AP. By reducing these variations, we conducted a more robust analysis, thereby we could perform analysis on a more reliable dataset. We demonstrated that the phylum abundance showed not significant shift in all case of AP progression, yet the diversity of species is reduced with the AP progression. This result indicating that the PAP and SAP may stem from an imbalance in the canal microbiota, causing a small number of bacteria to dominate and trigger infections, which may play a core microbial community of the disease.

It is important to note that we found simple variance analysis like PCA or Variance analysis consistently insufficient in providing adequate explanatory power, regardless of whether contamination was removed or not. Traditional variance analysis fell short in elucidating the differences across disease stages, which leads us to apply machine learning methods to explore and process the data. We used upsampling to handle imbalanced data and identified Logistic Regression (lb) as a suitable model through comparison. Different feature representation methods effectively reduced dimensionality while providing richer information, which have enhanced the transferability and repeatability of the conclusions drawn before (Bisanz et al., 2019). In this case, the adoption of techniques like PhILR and KO empowered us to unearth potential structured biological features within the ASV data, facilitating downstream analyses.

In contrast to traditional differential analysis, our machine learning approach enabled us to reverse-engineer the most decisive features. By employing advanced models, we pinpointed 14 ASVs that spanned different datasets, addressing the previous uncertainty regarding the importance of *E. faecalis* in SAP. Our analysis also shed light on less-recognized culprits, including *C. acnes* and *D. acidovorans*, which exhibited substantial increases in abundance exclusively in SAP. Both of these bacteria have been found to be associated with SAP in culture-based and PCR research (Anderson et al., 2012; Nardello et al., 2020; Pinheiro et al., 2022), but have long been overlooked in 16S rRNA-based studies. Our re-identification of them here underscores the necessity of unbiased meta-analyses that eliminate experimental errors. The core species identified through reverse engineering should not be understood as the sole cause of all cases of PAP or SAP. In many study, the presence of species like *E. faecalis* is not guaranteed. Understanding these core species should be framed as their alterations significantly increasing the probability of the disease or affecting its progression.

One remarkable finding is that *C. acnes*, *E. faecalis*, and *D. acidovorans* have been identified here as the most influential bacteria contributing to root canal failures. A common characteristic of these bacteria is their potential to form complex biofilms and carry antibiotic resistance genes (Pinheiro et al., 2022). Our research supports the conclusion that while *E. faecalis* is often associated with the formation of biofilms leading to apical periodontitis (AP), other species forming biofilms have been found in cases of SAP without *E. faecalis*. This suggests that the occurrence of SAP is not necessarily due to a specific species like *E. faecalis*, but rather more likely due to the presence of stubborn, difficult-to-remove biofilms. It is noteworthy that the importance of *C. acnes* and *D. acidovorans* may have been previously underestimated. The differential abundance might be attributed to the environmental conditions within necrotic dental pulps, which favor the growth of strict anaerobes. These anaerobes can ferment amino acids/peptides from necrotic pulp tissue and periradicular fluid (Mombelli and Décaillet, 2011). Conversely, the microbial composition might be influenced by changes in the root canal ecology post-treatment, leading to persistent infections (i.e., bacteria surviving from the primary infection) or secondary infections (i.e., invasion of oral microbes through coronal microleakages) (Siqueira et al., 2014).

Pathway analysis are consistent with the potential biofilm formation mentioned above. The enrichment of the phosphotransferase system (PTS) and the peptidoglycan biosynthesis pathway during the progression of apical periodontitis are aligned with previous reports of these pathways’ association with dental caries and suggest their involvement in the disease’s microbial community. Notably, these pathways are associated with sugar metabolism and biofilm/plaque formation, underscoring their relevance in the disease context (Chávez de Paz et al., 2007). Our research suggests a possibility that bacteria with high PTS activity and robust sugar metabolism may more easily form stubborn biofilms in the root canal environment. These biofilms could resist mechanical cleaning during primary apical periodontitis (PAP) treatment, leading to bacterial regrowth and the development of secondary apical periodontitis (SAP).

*Fusobacterium nucleatum* has been suggested as a keystone species in the development of periodontal disease. In the field of endodontics, *F. nucleatum* has been linked to primary infections, particularly in cases presenting clinical symptoms (Bouillaguet et al., 2018). Future transcriptomic analyses should explore the metabolism of Fusobacterium spp. in instances of acute endodontic infections.

Our findings offer new insights into the microbial landscape of AP and open avenues for precision-focused therapeutic interventions. By identifying microbial signatures and pathways associated with disease progression, we lay the foundation for targeted approaches to managing AP, potentially reducing the risk of treatment failures and improving patient outcomes. It is essential to acknowledge that the statistical power of our meta-analysis was constrained by the substantial amount of missing data in published studies. Efforts to minimize such gaps in future research are imperative.

## Data availability statement

The original contributions presented in the study are included in the article/**Supplementary Material**. Further inquiries can be directed to the corresponding authors.

## Author contributions

HL: Conceptualization, Funding acquisition, Investigation, Software, Writing – original draft, Writing – review & editing. JL: Conceptualization, Data curation, Formal analysis, Funding acquisition, Methodology, Writing – original draft, Writing – review & editing. JH: Resources, Visualization, Writing – original draft. JC: Supervision, Validation, Writing – review & editing. WZ: Funding acquisition, Project administration, Resources, Supervision, Writing – review & editing.
