# Supplementary material for: High-performing cross-dataset machine learning reveals robust microbiota alteration in secondary apical periodontitis
Source: Front Cell Infect Microbiol. 2024 Jun 21;14:1393108. doi: 10.3389/fcimb.2024.1393108 (PMC11224960; doi:10.3389/fcimb.2024.1393108)
Supplement: Supplementary file 1 [file DataSheet_1.pdf]

Supplementary Material

1 SUPPLEMENTARY DATA

All datasets analyzed in this study are available from public sources. A STORMS (Strengthening The Organizing and Reporting of Microbiome Studies) checklist is available at [doi: 10.5281/zenodo.10627821]. STORMS check list and other data could download from Zenodo.

2 SUPPLEMENTARY TABLES AND FIGURES

This document provides tables and figures that support our conclusions but were not displayed in the manuscript.

2.1 Figures

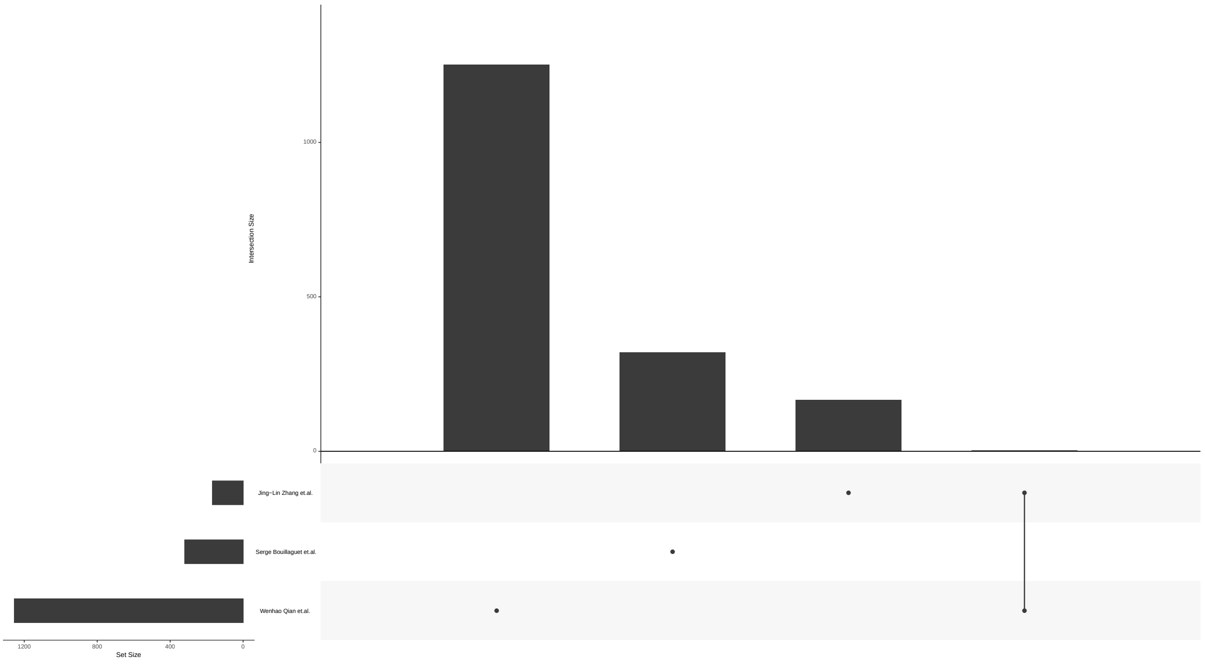

Figure S1. ASV counts and overlap of 3 data sources

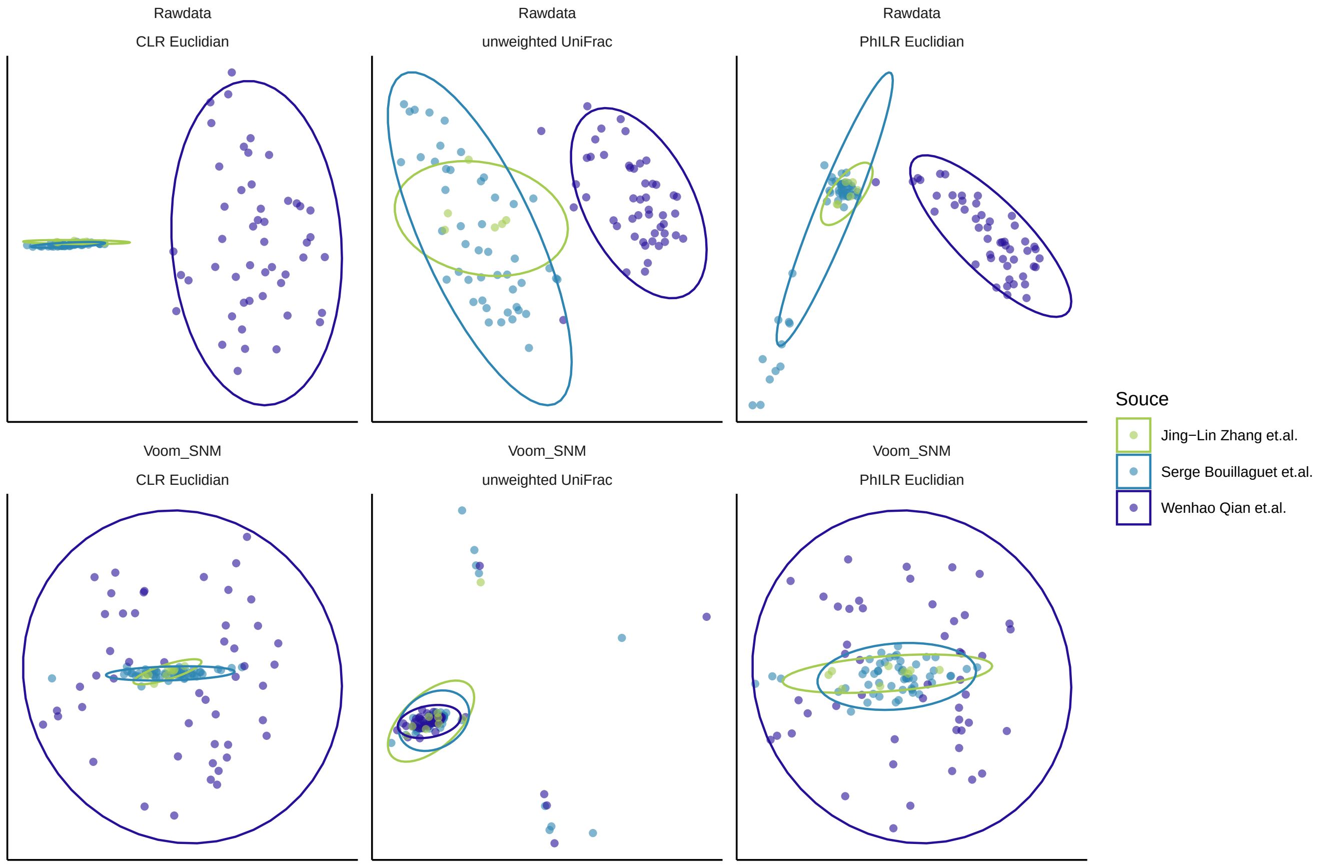

Figure S2. PCoA visualization of canal bacterial, colors refer to data source. Columns refers to beta-diversity distance used in PCoA

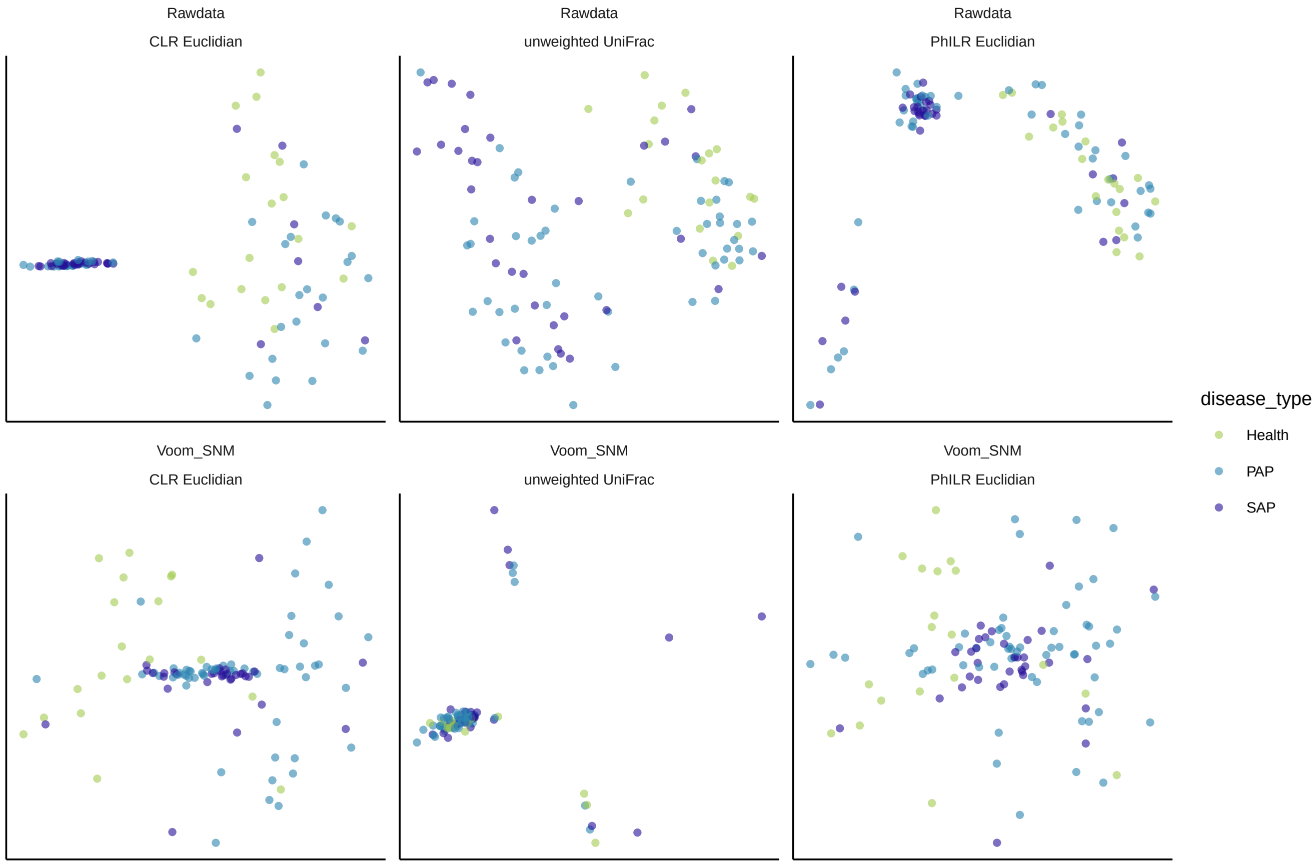

Figure S3. PCoA visualization of canal bacterial, colors refer to disease stage. Columns refers to beta-diversity distance used in PCoA

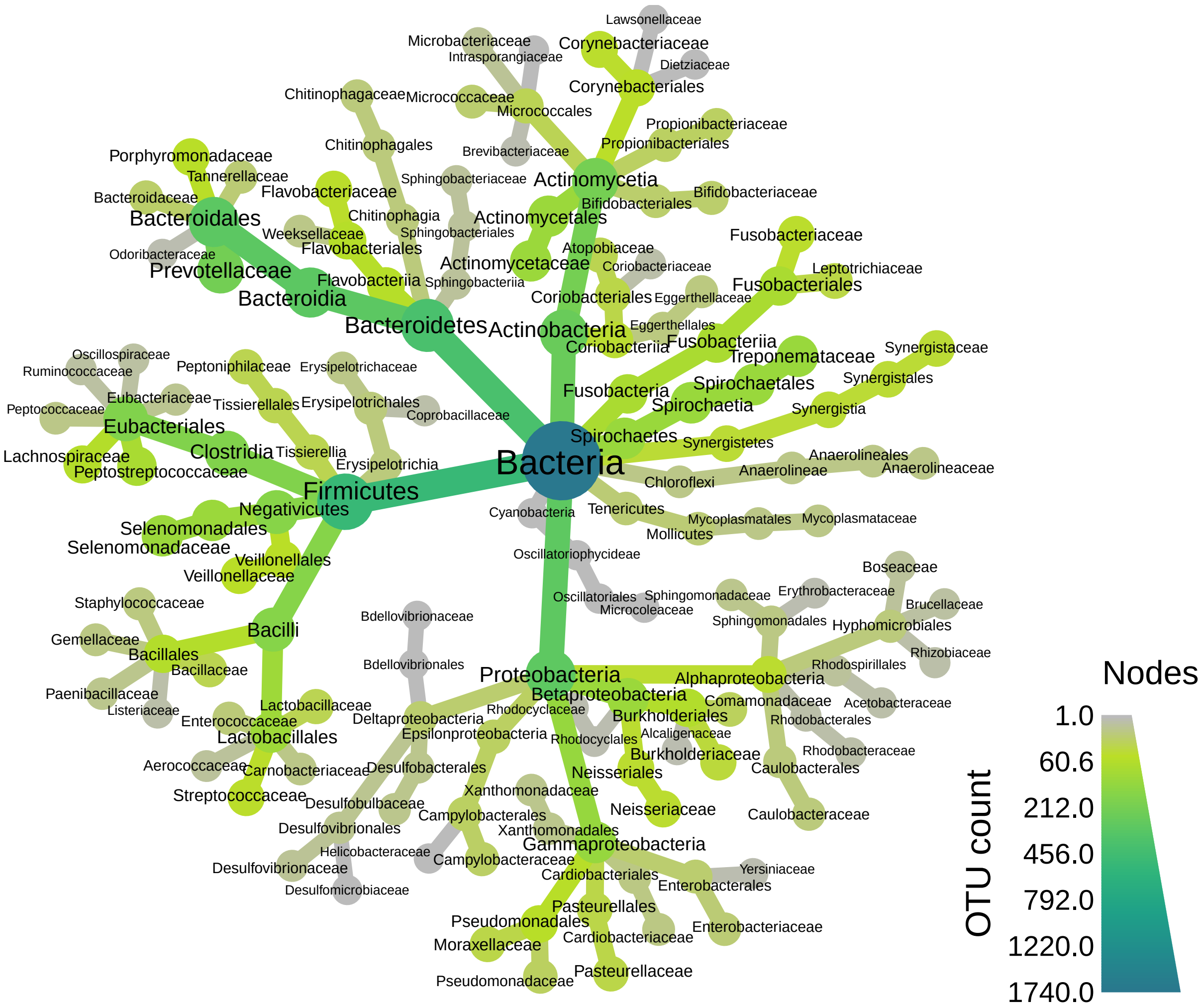

Figure S4. tree plot of SNM-corrected ASV data, color refers to counts

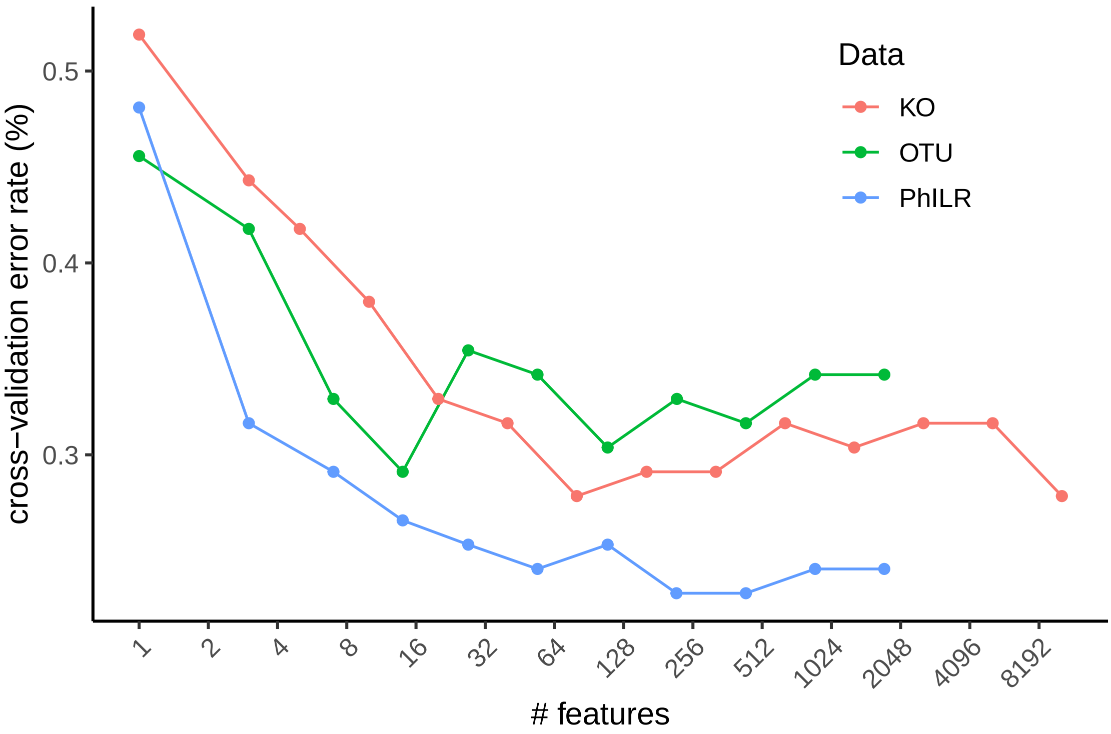

Figure S5. Cross-validation rate of different feature numbers of 3 kinds of data, showing that the KO / OTU / PhILR could reach lowest error rate trained with 80 / 14 / 200

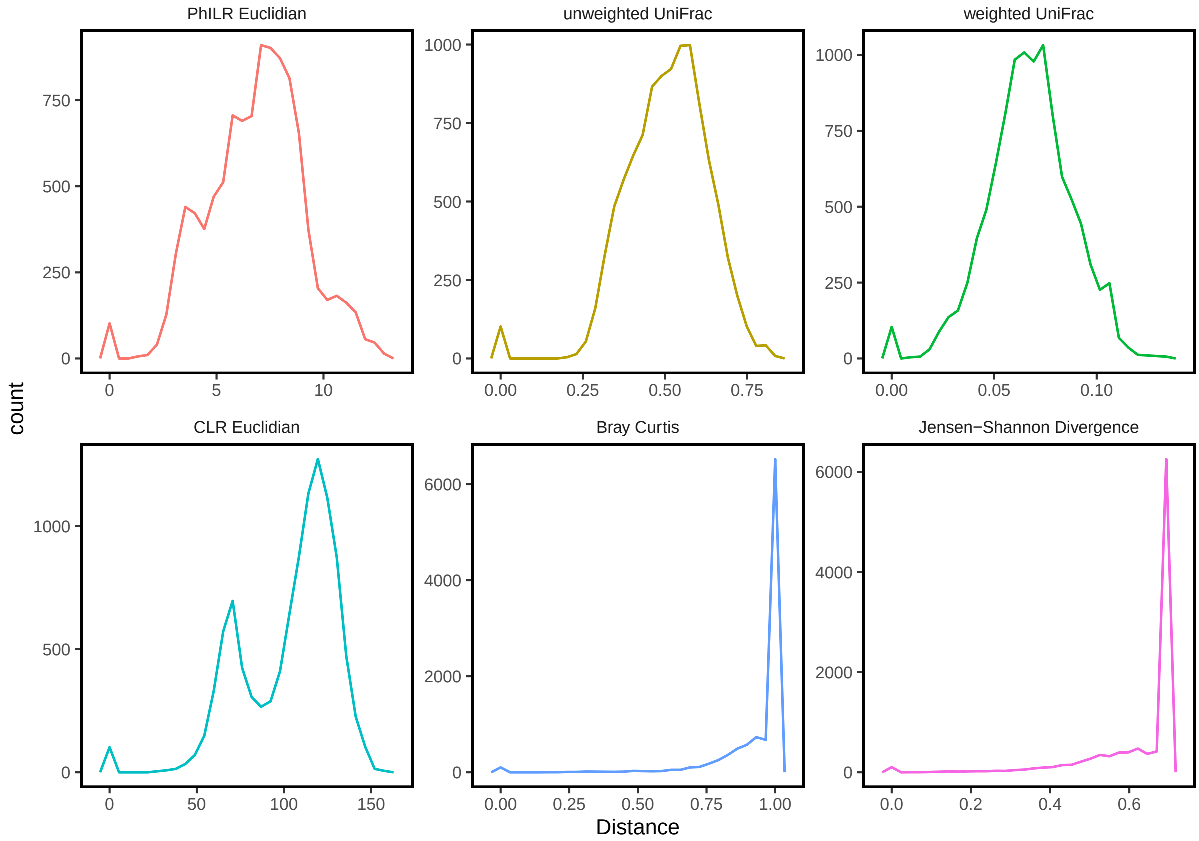

Figure S6. Distribution of different distances in Raw data

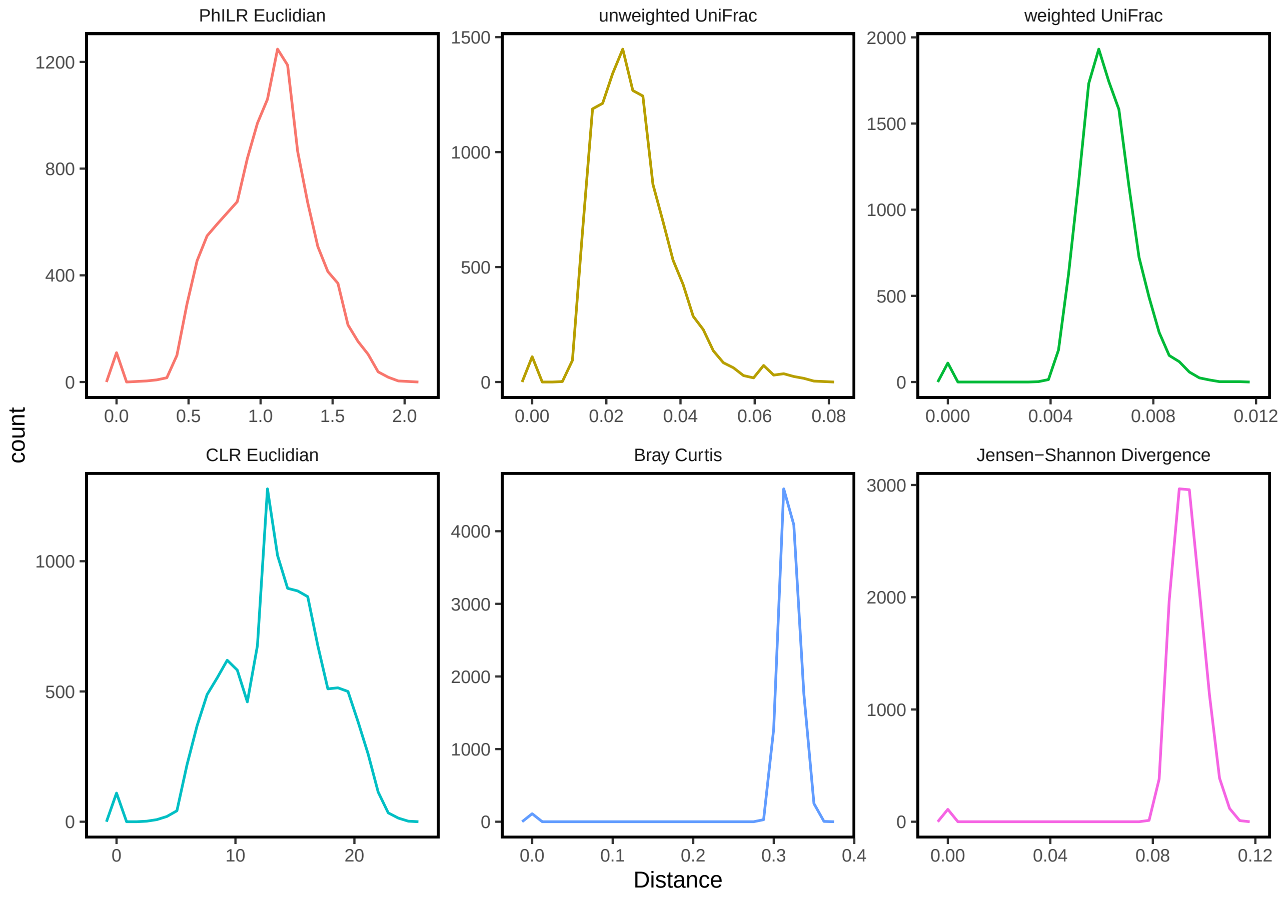

Figure S7. Distribution of different distances in SNM-corrected data

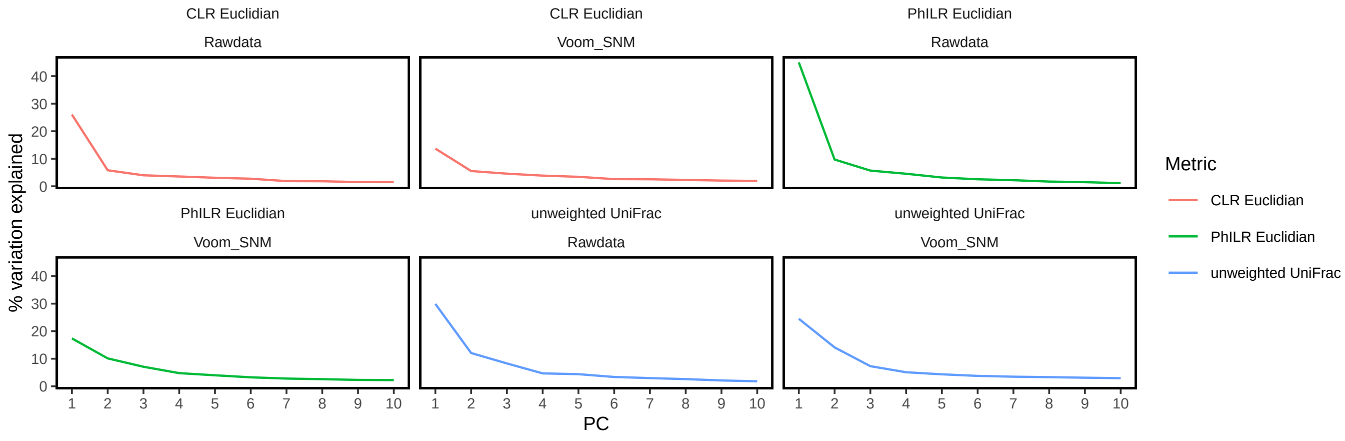

Figure S8. Scree Plots of different distances in raw data and voom-SNM corrected data

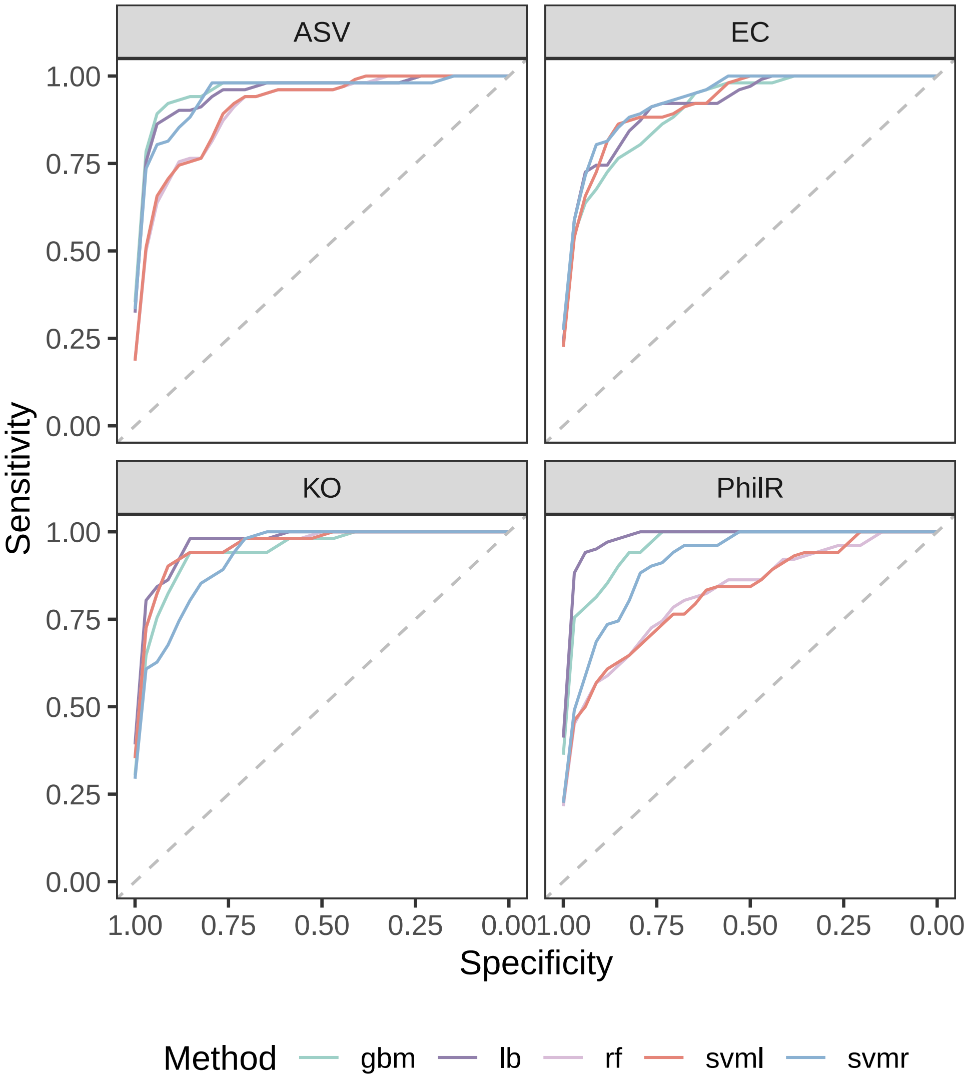

Figure S9. multiROC of different models in 4 kinds of dataset, lines are colored by training method

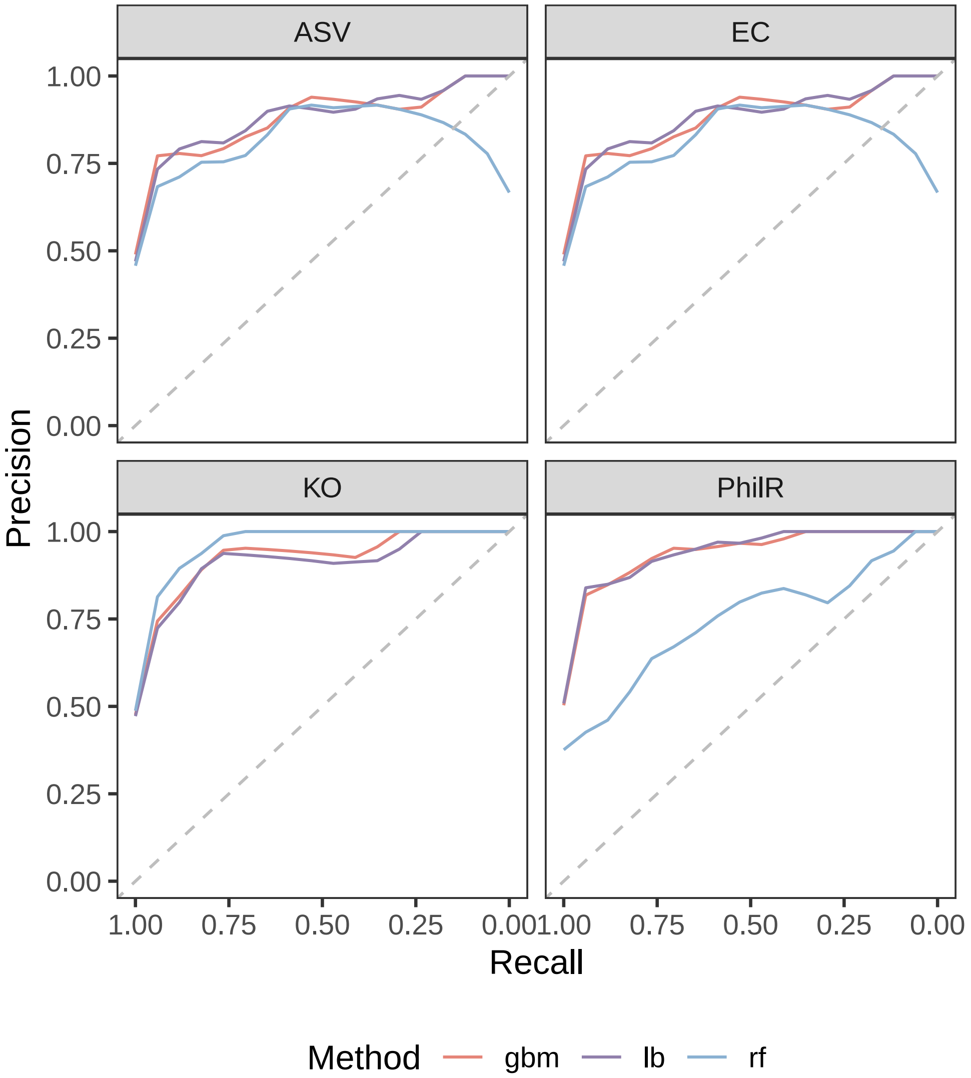

Figure S10. multiPR of different models in 4 kinds of dataset, lines are colored by training method, svml and svmr are not supported in this validation

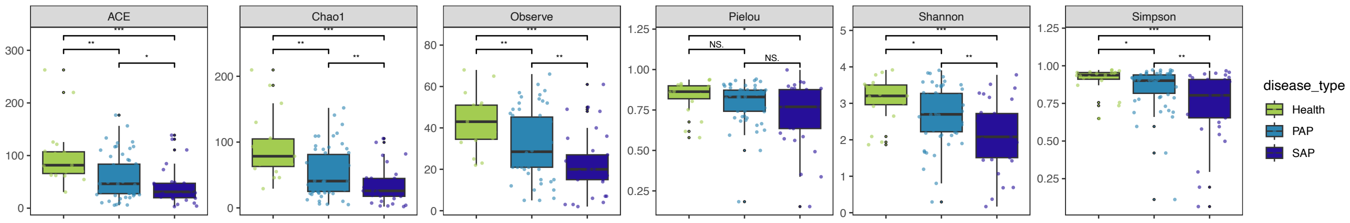

Figure S11. alpha diversity of each disease type, the signal indicating the statistic difference (*** $p \leq 0.001$; ** $0.001 < p \leq 0.01$; * $0.01 < p \leq 0.05$, NS, $0.5 < p$).

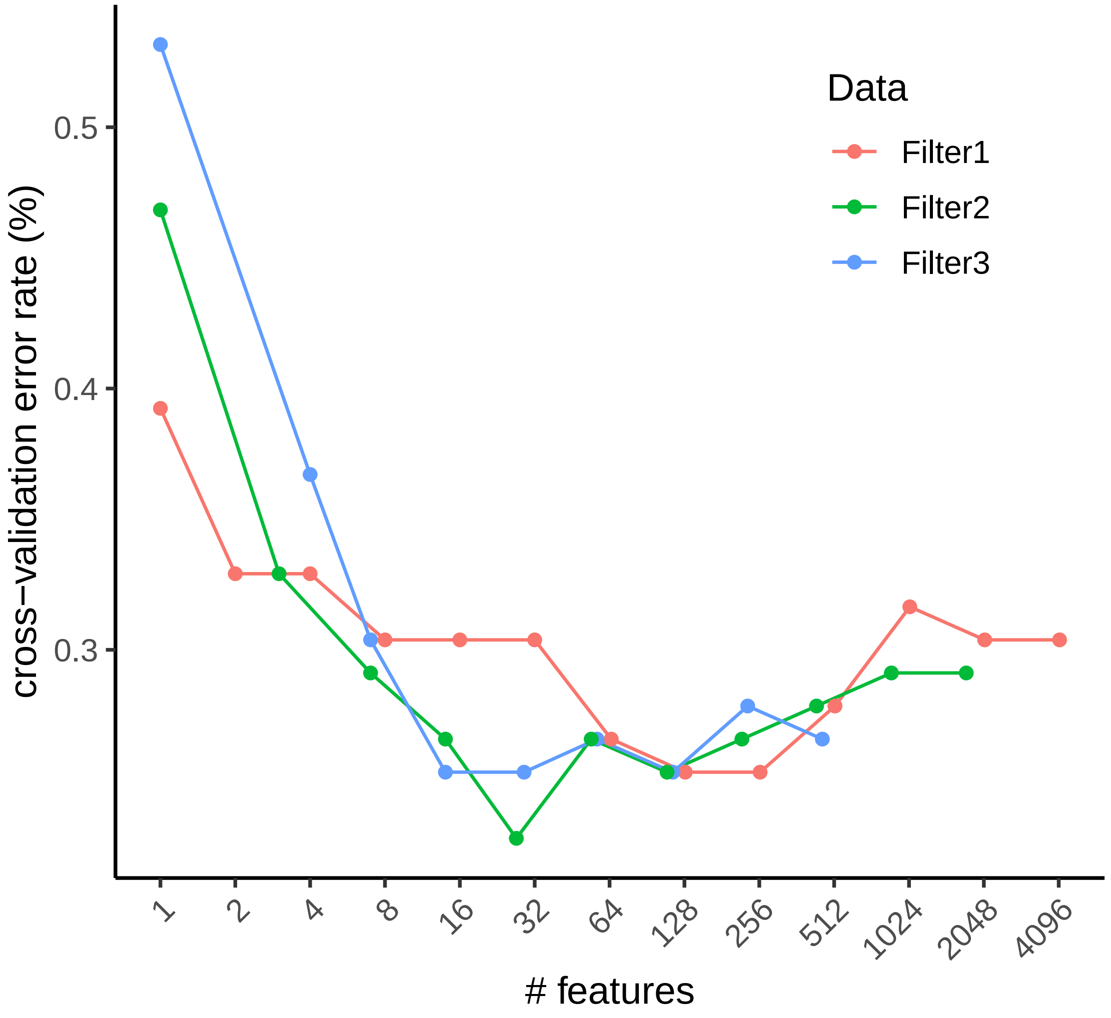

Figure S12. Cross validation error rate of different filtered data, filter1 refer to features presenting in at least 1 samples with at least a total of 10 reads, filter2 refer to features presenting in at least 3 samples with at least a total of 10 reads,filter3 refer to features presenting in at least 3 samples with at least a total of 500 reads.

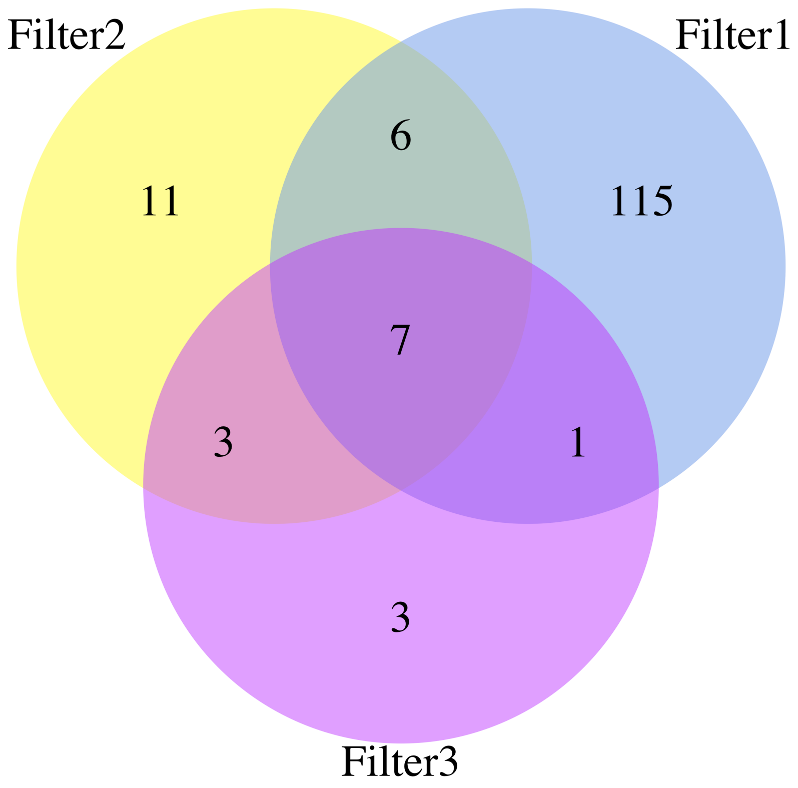

Figure S13. The overlap of key ASVs recognizing based on CV error from Random forest model with 3 filtered data. Filter1 refer to features presenting in at least 1 samples with at least a total of 10 reads, filter2 refer to features presenting in at least 3 samples with at least a total of 10 reads,filter3 refer to features presenting in at least 3 samples with at least a total of 500 reads. This Venn figure indicating the robustness of the result under varying data cleaning processes

2.2 Tables

Table S1. Adonis result of different distance metric and dataset

Metric	Term	Df	SumOfSqs	R2	F	Pr.F.
Rawdata-PhILR Euclidian	disease type	2	228.0436	0.1206	6.7883	0.0010
Rawdata-PhILR Euclidian	Residual	99	1662.8918	0.8794		
Rawdata-PhILR Euclidian	Total	101	1890.9354	1.0000		
Rawdata-CLR Euclidian	disease type	2	41026.8511	0.0789	4.2385	0.0010
Rawdata-CLR Euclidian	Residual	99	479142.5534	0.9211		
Rawdata-CLR Euclidian	Total	101	520169.4045	1.0000		
Rawdata-unweighted UniFrac	disease type	2	1.5870	0.1076	5.9710	0.0010
Rawdata-unweighted UniFrac	Residual	99	13.1566	0.8924		
Rawdata-unweighted UniFrac	Total	101	14.7437	1.0000		
Voom SNM-PhILR Euclidian	disease type	2	3.0551	0.0466	2.6166	0.0010
Voom SNM-PhILR Euclidian	Residual	107	62.4652	0.9534		
Voom SNM-PhILR Euclidian	Total	109	65.5203	1.0000		
Voom SNM-CLR Euclidian	disease type	2	629.8839	0.0567	3.2169	0.0010
Voom SNM-CLR Euclidian	Residual	107	10475.6843	0.9433		
Voom SNM-CLR Euclidian	Total	109	11105.5683	1.0000		
Voom SNM-unweighted UniFrac	disease type	2	0.0015	0.0316	1.7473	0.0180
Voom SNM-unweighted UniFrac	Residual	107	0.0457	0.9684		
Voom SNM-unweighted UniFrac	Total	109	0.0472	1.0000		

Table S2. Key EC pathways recognized from final model.

ID	Description	pvalue	p.adjust	qvalue
ec00051	Fructose and mannose metabolism	8.10842E-06	0.00081895	0.000716955
ec00020	Citrate cycle (TCA cycle)	0.000303287	0.010811916	0.009465356
ec00564	Glycerophospholipid metabolism	0.000321146	0.010811916	0.009465356
ec00730	Thiamine metabolism	0.000532161	0.013437055	0.01176355
ec00540	Lipopolysaccharide biosynthesis	0.000884018	0.017857155	0.015633153
ec00624	Polycyclic aromatic hydrocarbon degradation	0.002703118	0.045502484	0.039835421
ec00710	Carbon fixation in photosynthetic organisms	0.003498393	0.045879248	0.040165261
ec00720	Carbon fixation pathways in prokaryotes	0.004150411	0.045879248	0.040165261
ec00740	Riboflavin metabolism	0.00424284	0.045879248	0.040165261
ec00680	Methane metabolism	0.0045425	0.045879248	0.040165261

Table S3. Key KO pathways recognized from final model.

ID	Description	pvalue	p.adjust	qvalue
ko02060	Phosphotransferase system (PTS)	8.11E-06	0.00081895	0.000716955
ko00550	Peptidoglycan biosynthesis	0.000303287	0.010811916	0.009465356
ko00750	Vitamin B6 metabolism	0.000321146	0.010811916	0.009465356
ko00643	Styrene degradation	0.000532161	0.013437055	0.01176355
ko00626	Naphthalene degradation	0.000884018	0.017857155	0.015633153
ko00770	Pantothenate and CoA biosynthesis	0.002703118	0.045502484	0.039835421
ko00543	Exopolysaccharide biosynthesis	0.003498393	0.045879248	0.040165261

Table S4. F1 score of models trained with data without upsampling.

Methods	Health	PAP	SAP	Mean
RandomForest	0.6666667	0.7567568	0.6250000	0.6828078
SVM radical	0.5454545	0.6666667	0.0000000	0.4040404
SVM linear	0.6666667	0.7368421	0.5333333	0.645614
GBM	0.9090909	0.7272727	0.5555556	0.7306397
Logit boost	NA	NA	NA	NA

Table S5. Taxonomy of overlapped key ASVs recognized from filter1 and filter2 data

Phylum	Class	Order	Family	Genus	Species
Firmicutes	Bacilli	Lactobacillales	Enterococcaceae	Enterococcus	faecalis
Proteobacteria	Betaproteobacteria	Burkholderiales	Burkholderiaceae	Burkholderia	cepacia
Fusobacteria	Fusobacteriia	Fusobacteriales	Fusobacteriaceae	Fusobacterium	nucleatum
Synergistetes	Synergistia	Synergistales	Synergistaceae	Fretibacterium fastidiosum	
Bacteroidetes	Bacteroidia	Bacteroidales	Prevotellaceae	Prevotella	nigrescens
Firmicutes	Erysipelotrichia	Erysipelotrichales	Erysipelotrichaceae	Erysipelotrichaceae$_{[}G-1]$	bacterium$_{H}MT_{9}05$
Proteobacteria	Betaproteobacteria	Burkholderiales	Comamonadaceae	Delftia	acidovorans
Spirochaetes	Spirochaetia	Spirochaetales	Treponemataceae	Treponema	socranskii
Firmicutes	Erysipelotrichia	Erysipelotrichales	Erysipelotrichaceae	Solobacterium	moorei
Fusobacteria	Fusobacteriia	Fusobacteriales	Fusobacteriaceae	Fusobacterium	NA
